# Effect of methyl donors supplementation on performance, immune responses and anti-oxidant variables in broiler chicken fed diet without supplemental methionine

**DOI:** 10.5713/ab.20.0812

**Published:** 2021-06-24

**Authors:** Venkata Rama Rao Savaram, Venkata Lakshmi Narasimha Raju Mantena, Prakash Bhukya, Shyam Sunder Paul, Nagalakshmi Devanaboyina

**Affiliations:** 1 ICAR-Directorate of Poultry Research, Rajendranagar, Hyderabad-500030, Telangana, India; 2 College of Veterinary Science, PVNR Telangana Veterinary University, Hyderabad 500030, India

**Keywords:** Anti-oxidant Variables, Broiler Chicken, Immune Responses, Methionine, Methyl Donors, Performance

## Abstract

**Objective:**

Methionine (Met) is involved in methyl group transfer besides protein synthesis. As the availability is limited and cost is high for synthetic Met, reductions in its inclusion in broiler diet may be possible by supplementing the low Met diets with methyl donors (MD) like betaine (Bet), folic acid (FA), vitamin B12 (B12), and biotin (Bio). An experiment was conducted to study the effects of supplementing the MD on performance (average daily gain [ADG], daily feed intake, feed efficiency [FE]), anti-oxidant variables, immune responses and serum protein concentration in broilers fed sub-optimal concentrations of dietary Met.

**Methods:**

Maize-soybean meal diet was used as control (CD). Different MD like Bet (0.2%), B12 (0.1 mg), FA (4 mg), or Bio (1.5 mg/kg) were supplemented to basal diet (BD) having no supplemental Met. The BD without MD was kept for comparison. Each diet was fed *ad libitum* to 10 replicates of 25 chicks in each from 1 to 42 d of age.

**Results:**

At the end of experiment, the ADG in MD group was higher than BD and lower than CD. The FE improved with FA or Bet compared to the BD. Breast meat weight was higher in Bet compared to the BD, while it was intermediate between BD and CD in other groups. The lipid peroxidation reduced with Bio, B12, or Bet, while the glutathione peroxidase activity improved with Bio or B12 compared to the BD. Lymphocyte proliferation improved with Bet compared to the BD. The serum protein concentrations increased with FA, Bio, or Bet compared to those fed BD.

**Conclusion:**

It can be concluded that the ADG can be improved partially with supplementation of MD while the FE improved with FA or Bet. Some MD also reduced the stress indices and improved immune responses compared to the BD fed broilers.

## INTRODUCTION

Methionine (Met) is an important amino acid which is essential for the proper development of muscle [1] and immune response [2] in chicken. Typical maize-soybean meal based broiler diet is deficient in Met and is supplemented with crystalline DL-Met to meet the bird’s requirement. Another important function of Met in biological systems is the methyl group sparing activity [3,4]. Met also plays an important role in the trans-sulfuration pathway, where it serves as a precursor for the synthesis of cysteine and also acts as an intermediate in the transmethylation pathway [5]. The non-availability and higher cost of synthetic Met often force the poultry feed manufacturers to look for alternate sources for the amino acid. Florou-Paneri et al [6] showed that between 30% and 80% of the supplemental Met can be substituted with methyl donors (MD) without negative effects on the performance of chickens.

The nutrients like betaine (Bet), vitamin B12 (B12), folic acid (FA) and biotin are also involved in the single carbon (methyl) group transfer. Methyl groups are necessary for the synthesis of various essential compounds, such as Met, carnitine, creatine, phospholipids, adrenal hormones, RNA, and DNA [7]. Supplementation of MD at higher concentrations in the diet helps in transmethylation activity and permits Met to be directed for protein synthesis and may partially reduce the dietary need of this amino acid for MD function [8].

Betaine is a natural MD, which donates the methyl group to homocysteine for Met synthesis [9]. On the molecular weight basis, Bet contains about 3.75 times the methyl groups compared to Met. Vitamin B12 plays an important role in the homocystein metabolism and immune system functions. Similarly, FA acts as a cofactor in the methylation reactions, particularly in the synthesis of Met [10]. FA in the form of tetrahydrofolate methylates the homocysteine to Met. Jing et al [11] observed reduced inflammatory reactions, induced by lipopolysaccharide challenge, with higher dietary concentration of FA (4 mg/kg) in egg laying chicken. Biotin supplementation (200 to 400 μg/kg) was reported to improve the performance of broiler chicken [12].

Finding a viable alternative for the expensive synthetic Met in broiler diets may aid in partially reducing dependence on DL Met and maintaing optimum production during the amino acid scarcity. Dietary Met plays an important role in immune responses [2] and anti-oxidant status and hence reduction of the Met levels in diet would influence both the vital functions [11]. It is essential to evaluate anti-oxidant and immune responses in broilers fed low-Met diets supplemented with various MD. Information about the beneficial effects of MD on broilers fed diets devoid of supplemental Met is not available in the literature. Therefore, an experiment was conducted to study the effects of supplementing higher concentrations of various MD (Bet, B12, FA, biotin) on performance, anti-oxidant variables and immune responses in commercial broilers fed diets with sub-optimal concentrations of Met.

## MATERIALS AND METHODS

### Birds and management

Commercial male broiler (Cobb 400) chicks (n = 1,500) were randomly and equally distributed into 6 dietary groups having 10 replicates with 25 chicks per replicate. The birds were reared in deep litter floor pens (4′6″×6′) from d 1 to 42 d of age in an open sided poultry house. Artificial heat was provided to brood the chicks at 37°C±1°C up to 7 d of age, which was gradually reduced to 27°C±1°C by 21 d of age, after which, the broilers were reared at ambient temperature (25.3°C to 34.2°C). Florescent bulbs were used to provide 22 h light and 2 h darkness in a day from d 22 till the end of experiment. The birds were vaccinated against Newcastle (Lasota) disease (ND) on d 5 and 21 and Infectious bursal disease on 11 and 28 d of age. The experiment was conducted by following the guidelines of the Institute Animal Ethics Committee (IAEC/DPR/17/1: 21/10/2017).

### Diets

Maize and soybean meal based control diets (CD) having 2,925, 3,050, and 3,100 kcal/kg ME and 22%, 20%, and 18.5% crude protein (CP)/kg, respectively for pre-starter (1 to 14 d), starter (15 to 28 d), and finisher (29 to 42 d of age) phases were prepared (Table 1). Crystalline Met (DL-Met) was supplemented to the CD to meet the Met requirement of the broiler strain (0.55%, 0.48%, and 0.45%, respectively in pre-starter, starter, and finisher diets). A basal diet (BD) with maize – soybean meal was prepared with nutrient composition similar to that of CD without supplementation of DL-Met and other MD tested. Both the CD and the BD were analysed [7] for lysine and Met. Calculated concentrations of Met in the BD were 0.328%, 0.303%, and 0.285%, respectively in pre-starter, starter, and finisher phases. The BD was supplemented independently with higher concentrations of biotin (1.5 mg/kg), B12 (0.1 mg/kg), Bet anhydrous (0.2% in diet) or FA (4 mg/kg). The selected concentrations of MD were based on the values either suggested or higher than those recommended in the literature [13–15]. FA, biotin, and B12 were procured from BASF and Bet was from Danisco. Each diet was allotted to 10 replicates of 25 chicks each by following completely randomized design and fed *ad libitum* from d one to 42 d of age.

### Parameters recorded

Body weight and feed intake (FI) per replicate were recorded at the end of each phase. Average daily gain (ADG) and daily feed intake (DFI) were calculated for each replicate. Feed efficiency (FE) was calculated as FI/body weight again (BWG).

Blood samples (about 2 mL) were collected from the brachial vein of one bird in each replicate at 42 d of age to analyze the concentrations of total protein (TP) and albumen in serum utilizing the diagnostic kits (Product No 72111 and 72131, respectively, M/S Qualigens India, Mumbai, India).

At 43 d of age, one bird from each replicate having the body weight nearer to the average of the replicate (±5%) was slaughtered by cervical dislocation to study the carcass variables. The ready-to-cook (RTC) yield (without liver, gizzard, and heart) and weights of liver, abdominal fat and breast were recorded and expressed as g/kg pre-slaughter live weight of the respective bird.

The oxidative parameters like lipid peroxidation (LP) and the activities of anti-oxidative enzymes like red blood cells (RBC) catalase (RBCC) and glutathione peroxidase (GSHPx) in blood were measured. About two mL of blood was drawn from the brachial vein of one bird per replicate at 43 d of age into a centrifuge tube containing citrate buffer (1.5 mL/10 mL blood) for erythrocyte separation and antioxidant enzyme estimation. The blood samples were centrifuged at 500×g for 15 min at 4°C to separate buffy coat (WBC) and form erythrocyte pellet. The erythrocytes were washed thrice with PBS (pH 7.4). The packed RBC obtained was mixed with an equal volume of PBS and then diluted as per the requirement with distilled water.

The LP was estimated by quantifying malonyl dialdehyde (MDA), a secondary product of LP. The MDA reacts with 2-thiobarbituric acid to form a trimethine colored substance (pink chromogen), which was extracted into butanol. The color intensity was measured at 548 nm. The LP activity in the erythrocytes was expressed in nmol MDA/mg protein [16].

The enzyme catalase decomposes H_2_O_2_ and the rate of decomposition as measured in terms of reduction in absorbance is indicative of the enzyme activity in the serum sample [17]. The RBCC activity was expressed as units per g of hemoglobin (Hb) after estimating Hb concentration in the haemolysate.

The activity of GSHPx was estimated following the method of Paglia and Valantine [18].

The effect of supplementing MD on cell mediated immunity (CMI) (*in vitro* lymphocyte proliferation ratio [LPR]) and humoral immune (HI) responses (antibody response against ND vaccine) was studied. The blood samples were collected at 20 d of age from one bird per replicate in all the treatments to study the CMI and HI responses. At 20 d of age, 2 mL of blood was collected from one bird per replicate and the antibody titres in sera against ND virus were measured [19] by haemagglutination test.

The difference between the *in vitro* proliferation of lymphocytes with and without the stimulant (concanavalin A, Con A) was expressed as the ratio. The LPR was assayed using MTT tetrazodium salt (3-(4,5-dimethylthiazol-2-yl)-2, 5-diphenyl tetrazolium bromide) [20]. About 2 mL of blood was collected from the brachial vein of the bird in a centrifuge tube containing heparin disodium salt (5 mg). One bird from each replicate was used to collect blood samples at 20 d of age. The un-clotted blood sample was layered gently over histopaque 1077 (Sigma, Mumbai, India) and centrifuged at 500×g for 20 minutes at 4°C. The cellular band at the interface was collected and transferred to another tube and washed 3 times with RPMI 1640 medium (AL 028A, Himedia, India). The viable cells were counted by using the trypan blue dye exclusion method and the cell concentration was adjusted to 1×10^7^ cells/mL of RPMI 1640 medium. These cells (10^5^ purified lymphocytes) were used to measure lymphocyte proliferation by adding 10 μL of suspension to each well of a 96-well flat bottom sterile tissue culture plate. Con A (0.9 μg in 150 μL RPMI/well) was used as the stimulant for lymphocyte proliferation. The plate was incubated at 37°C and 5% CO_2_ concentration for 69 h in a humid atmosphere, then 20 μL MTT (10 mg/mL) was added to each well and the plate was re-incubated for 3 h. At 72 h, 100 μL of 4% 1 N HCl – iso propanol was added to each well and mixed thoroughly with a micropipette to dissolve the formazin crystals, which gave a deep purple color. The color intensity was measured in an ELISA reader (V.200.1, μ Quant, Biotek Instruments, Inc., Winooski, VT, USA) at 550 nm. The LPR was calculated as optical density (OD) of well with Con A – OD of well without Con A)/OD of well without Con A.

### Statistical analysis

The mean of a pen was considered as an experimental unit for performance data and the value of the individual bird was considered for slaughter variables, serum parameters and immune responses for statistical analysis with one-way of analysis of variance [21]. The means were compared with Tukeys test to find the significant difference between treatment effects.

## RESULTS

### Performance

The ADG was significantly reduced in broilers fed the BD having sub-optimal concentrations of Met compared to those fed the CD (Table 2). During the pre-starter phase, the ADG was significantly (p<0.05) improved with supplementation of all MD compared to those fed the BD, while the weight gain in B12 or Bet supplemented groups was similar to the CD fed broilers. Supplementation of the MD except Bet to the BD did not improve the weight gain during starter phase, while the ADG in Bet group was significantly higher than the BD group. The ADG over the entire experiment (1 to 42 d) was significantly higher in MD supplemented groups compared to the BD, but the growth was significantly inferior to the CD.

The DFI was significantly (p<0.05) reduced in broilers fed the BD compared to the CD fed group during all phases except the finisher phase, while the DFI was not affected by the treatments employed in the current study. The DFI during pre-starter phase was improved with the MD supplementation similar to those fed the CD. Similarly, such an improvement was observed during starter phase with all the MD, except FA. Supplementation of Bet or biotin to the BD improved the DFI similar to the CD during the entire experiment period (1 to 42 d), while the DFI in other MD fed broiler was intermediate to the CD and BD fed broilers.

The FE was not affected (p>0.05) by the treatments during the pre-starter and finisher phases, while the FE was lower (p<0.05) in the groups fed the low-Met BD irrespective of MD supplementation during the starter phase (Table 2). Similarly, the FE was significantly lower in BD fed group compared to the CD group during the overall experimental period (1 to 42 d). The feed efficiency did not improve with supplementation of B12 or biotin compared to those fed the BD. However, the feed efficiency in broilers fed FA or Bet improved (p<0.05), which were in between the CD and BD fed broilers.

### Slaughter variables

The RTC yield and relative weight of abdominal fat were not affected (p>0.05) by the dietary Met level, or supplementation of MD to low-Met BD (Table 3). The relative weight of breast reduced numerically (13.2%) in broilers fed the BD compared to those fed the CD. The breast weight in FA, biotin or B12 supplemented groups was similar (p< 0.05) to those fed the CD. The breast meat weight in Bet supplemented groups was significantly (p<0.05) higher than those fed the BD.

### Serum anti-oxidant variables

#### Lipid peroxidation

The serum LP was significantly (p<0.05) higher in broilers fed the BD compared to those fed the CD (Table 4). The LP in biotin, B12, and Bet groups was significantly (p<0.05) reduced compared to those fed the BD. The LP in B12 or Bet groups was similar to those fed the CD, while the LP in FA group was similar to the BD group.

#### Glutathione peroxidase

The activity of GSHPx showed a reduction (p<0.05) in the low-Met BD fed group compared to the CD group. The enzyme activity in biotin or B12 groups was significantly (p<0.05) higher than those fed the BD and was similar to the CD group (Table 4). The anti-oxidant enzyme activity in the Bet or FA groups did not improve compared to the BD fed group.

### Red blood cells catalase

The activity of RBCC reduced significantly (p<0.05) in broilers fed the low-Met BD compared to the CD fed group. Supplementation of biotin or Bet significantly (p<0.05) improved activity of the anti-oxidant enzyme compared to those fed the BD. The enzyme activity in FA or B12 fed groups did not improve compared to those fed the BD.

### Immune responses

The anti-body titres against ND vaccine did not differ (p>0.05) due to the treatments employed (Table 5). The LPR reduced significantly (p<0.05) in groups fed the low-Met BD compared to the control group. The LPR in groups fed FA, biotin, or B12 did not differ compared to those fed the BD, whereas the LPR improved significantly in Bet supplemented groups compared to the BD fed broilers.

### Serum protein fractions

Feeding of diets having sub-optimal concentration of Met (BD) significantly (p<0.05) reduced the concentrations of TP and albumin compared to those fed the CD (Table 5). The concentration of TP improved significantly (p<0.05) in broilers fed the FA, biotin or Bet supplemented diets compared to the BD fed birds. The TP in B12 supplemented group was similar to the BD fed group. Similarly, the albumin concentration in all the MD supplemented groups improved similar to those fed the CD.

## DISCUSSION

### Performance

Supplementation of different MD to the low-Met BD improved the ADG of broilers compared to those fed the BD during the pre-starter phase. Similar improvement in BWG with Bet supplementation during the starter phase and all MD during the entire experimental period was observed. Significant improvement in DFI during the entire experimental period was observed in broilers fed Bet and Bio compared to those fed the BD and the FI in the latter two groups was similar to the CD fed broilers. Thus, the results indicate the Met-sparing function of FA, biotin, B12 or Bet at the higher concentration in diets having sub-optimal concentrations of Met. The results of the current study are in line with the findings of Mahmoudi et al [22], who reported that dietary Met could be replaced up to 20% with Bet supplementation. Similarly, dietary supplementation of B12 [13], biotin [12], FA [23] or Bet [8,24,25] was reported to improve the performance of broilers fed the diets containing either adequate or sub-optimal concentrations of Met.

The overall FE (1 to 42 d) in the groups fed FA or Bet was similar to those fed the CD. Similar to these observations, improvement in FE was observed with supplementation of Bet [8,16] or FA [26] to diet containing sub-optimal concentrations of Met.

Though the performance of broilers fed the low-Met BD supplemented with MD was significantly higher than the BD, the weight gain was not similar to those fed the CD, which suggested that the MD could not able to completely alleviate the negative effects of feeding diets without supplemental Met. The marginal improvement in performance of broilers fed the low Met BD with MD supplementation could be due to the Met-sparing effect of the MD tested. Since the MD tested play an important role in the transmethylation cycle, inclusion of higher concentrations of these MD could able to improve the broiler performance probably by sparing the MD function of Met. Chamruspollert et al [3] indicated that one of the major functions of Met in biological systems is the methyl group-sparing activity apart from protein synthesis. As all the vitamins and Bet are potent methyl group contributors, these MD could able to support the growth in broilers fed the sub-optimal levels of Met in diet. Majority of MD plays key role in the conversion of homocysteine to Met by donating methyl group through transmethylation metabolic pathways.

Contrary to the present findings, literature [24,27] reported lack of improvement in broiler performance with MD (Bet) supplementation. The lower level (400 mg/kg) of Bet [27] or Met (13.4 and 13.6 g/kg CP, respectively, in starter and grower) in the basal diet [24] used by the respective authors might be responsible for the absence of response to Bet supplementation. The data thus suggested the importance of maintaining a minimum concentration of Met in the basal diet to elicit any response to Bet supplementation. Sun et al [4] reported that Met can be replaced up to 25% with Bet without adversely affecting the production performance of the broilers, indicating that up to 25% of dietary Met is involved in methylation reactions in broilers. In the current study, the Met deficiency in BD was higher (41.9%, 36.9%, and 34.5%, in pre-starter, starter and finisher phases, respectively) than the 25% as indicated in the literature. The beneficial effects of MD on performance [1] and carcass attributes [9] were reported to depend on the concentration of Met in diet. At higher concentrations of dietary Met (30.1, 27.6, and 28.2 g/kg CP), Waldroup et al [1] observed no improvement in body weight and other performance variables with Bet supplementation. Similarly, in our previous study [8], beneficial effects of Bet supplementation on performance and carcass variables were observed only at the sub-optimal concentrations of Met (15 g/kg CP), but not at the higher concentrations (22 and 24 g/kg CP) of the amino acid in diet.

Though the ADG was improved with majority of the MD tested, the FE was not affected with biotin and B12 supplementation. From the literature [27], it is clear that MD cannot substitute Met for protein synthesis, but can spare MD activity. The lack of response in FE in broilers fed BD with different MD could be due to the severe deficiency of Met in the BD, which was probably lower than the minimum required concentration of Met for the protein synthesis function and therefore, supplementation of biotin or B12 could not able to improve the feed efficiency. Similar to these observations, Rostagno and Pack [24] and our previous study [8] also did not report improvement in broiler performance with MD (Bet) supplementation to low-Met basal diet (13.4 and 13.6 g/kg CP). It is worth noting that in both the current and our previous studies, crystalline Met was not added to the maize-soybean meal based basal diets. Therefore, the data thus suggested the importance of maintaining a minimum level of Met in the basal diet to elicit any response in performance to the MD supplementation.

The improved ADG of broiler with MD supplementation to the BD could be due to increased nutrient digestibility and the important role of MD in many reactions including DNA and RNA methylation and protein synthesis, besides the methyl groups sparing function. Though nutrient digestibility was not assessed in the current study, Bet supplementation was reported to improve the digestibility of CP [28], crude fibre and ether extract [29].

### Carcass variables

The lowest relative weight of breast meat was observed in the broilers fed low-Met BD and the breast meat weight in Bet-supplemented groups was significantly higher than those fed the BD and the weight of breast meat in other MD fed groups was similar to those fed the CD. Similarly, other investigators have also demonstrated improved breast meat yield with Bet supplementation to the standard diet [1,25] or diets deficit in Met [8]. B12 supplementation was also reported to improve the breast meat weight [13]. Contrary to the current findings, Waldroup and Fritts [30] did not find improvement in breast meat yield in broilers fed the diet supplemented with Bet. The lack of response could be due to the relatively lower inclusion level of Bet (0.1%) compared to the current study (0.2%). Though the breast weight was affected with MD supplementation to the low Met BD, the abdominal fat weight was not influenced by the dietary treatments. Similar to the current findings, McDevitt et al [31] and our previous study [8] also reported that Bet is ineffective in influencing the deposition of fat in abdominal area.

The relative weight of liver increased significantly in broilers fed Met-deficit diet (BD) compared to those fed the CD. MD supplementation significantly reduced the liver weight, which could be due to the increased fat mobilization from liver with MD supplementation.

### Anti-oxidant variables

The increase in LP and reduction in the activities of GSHPx and RBC catalase in the serum of broilers fed the BD compared to those fed the CD suggested that the reduction of dietary Met concentration increased oxidative stress in broilers. Oxidation of lipid in body tissue produces MDA, which is the main aldehyde derivative of peroxidation, and it is a by-product of the lipid peroxidation processes [32]. As Met is an important component of glutathione, which helps in scavenging the free radicles and reducing the ill effects of oxidative stress, the deficiency of Met (BD fed group) increased the LP and reduced the activities of anti-oxidant enzymes (GSHPx and RBCC) in broiler. Supplementation of biotin, B12, or Bet significantly reduced the LP, whereas biotin or B12 increased the activity of GSHPx and similarly, biotin or Bet improved the activity of RBCC compared to the low-Met BD fed broilers. Reduced LP with MD supplementation could be due to the Met-sparing function of MD or the direct role of MD in the synthesis of compounds required for anti-oxidant activity [33].

Bet is one of the MD, which was studied extensively as a partial substitute for Met in terms of performance and oxidative responses. Alirezaei et al [9] suggested that the reduced oxidative stress with Bet supplementation is mediated by restoring S-adenosyl Met, which contributes the substrate needed for the synthesis of glutathione. Similar to the current findings, the above authors [34] reported reduced oxidative stress with Bet supplementation in broilers fed the low-Met diet. Reduction in breast muscle oxidative process with Bet supplementation is one of the mechanisms of reduced LP in the serum of broilers fed MD supplemented diets [16]. Similar to the current findings, significant reduction in LP was reported with Bet supplementation in broiler diet [9,16].

Among the MD, FA did not affect the LP or the activities of anti-oxidant enzymes, which is contrary to the findings of Sahin et al [35], who observed significant reduction in the serum and tissue MDA (LP) and other stress indices (homocysteine and ACTH) in heat stressed Japanese quails with FA supplementation (1 mg/kg) in diet. The contradicting results may be due to the heat stress induced by Sahin et al [35] and the use of lower concentrations of FA in their study.

### Immune responses

The cell mediated immune response (LPR) was significantly reduced in broilers fed the Met-deficit diet compared to those fed the CD. Among the MD tested, Bet significantly improved the CMI response, higher than those fed the BD, which implies that Bet improved the CMI response in broilers fed the Met-deficit diet. These results are in line with our previous findings [8] and other reports [25]. In our previous report, significant improvement in LPR with Bet supplementation was observed with diets containing sub-optimal concentrations of Met (15 g/kg CP), but not in the groups fed the adequate concentrations of the amino acid. The increased LPR with Bet supplementation might be due to the increased digestibility and utilization of Met [36] and other nutrients like carotenoids, lysine, protein and fat [37], which are known to influence immune responses [38]. Increased LPR with Bet supplementation might also be due to its role in enhancing phagocytosis and the release of inflammatory cytokines [39] and nitric oxide by heterophils and macrophages and increasing the chemotactic effects of monocytes [40].

### Serum protein fractions

In general, the concentration of serum albumin was lower than the standard values in all the treatment groups. The reasons for such lower values are not known. The concentrations of TP and albumin in serum reduced in broilers fed the BD compared to those fed Met adequate CD. Among the MD, FA, biotin or Bet supplementation improved the serum concentration of TP, whereas the serum albumin concentration increased in the FA or Bet supplemented groups. The increased nutrient utilization, particularly of the dietary protein reported with the MD supplementation [37,36] might have resulted in the improved performance and feed efficiency in broilers fed the sub-optimal concentrations of Met [30]. The increased concentrations of TP and albumin in the serum of broilers fed the MD supplemented diet also suggested the favorable effects of MD on nutrient utilization, bird performance and cell mediated immune response as observed in the current study. Similarly, FA supplementation was reported to improve protein metabolism [41,42] and elevate the concentrations of TP, globulin, and albumin in plasma or serum [41,42].

## CONCLUSION

The study indicated that supplementation of B12 (1.5 mg/kg), FA (4 mg/kg), biotin (1.5 mg/kg), or Bet (0.2%) partially improved the weight gain and such improvement in feed efficiency was observed with FA or Bet supplementation in broilers fed maize-soybean based diets without supplemental Met. Methyl donors also reduced stress indices (reduced LP and increased activity of anti-oxidant enzymes). The performance improvement may partly be due to the improvement in protein utilization as evident by increase in protein concentration in the serum and or increased FI with MDs supplementation in broilers fed diets supplemented with MD.

## Figures and Tables

**Table 1:** Ingredient and nutrient composition of control diets

Items	Pre starter (1 to 14 d)	Starter (15 to 28 d)	Finisher (29 to 42 d)
Ingredient (g/kg)			
Maize	553.03	596.09	639.82
Soybean meal	387	334	292
Vegetable oil	21.5	33.2	32.8
Common salt	4	4	4
Sodium bi-carbonate	1	1	1
Dicalcium phosphate	18.8	17.5	16.6
Shell grit	7.2	7.2	6.9
DL-methionine	2.27	1.81	1.68
Premix^1)^	5.2	5.2	5.2
Nutrient %			
Metabolizable energy (kcal/kg)^2)^	2,925	3,050	3,100
Crude protein^3)^	22	20	18.5
Lysine^2)^	1.21	1.08	0.95
Lysine^3)^	1.301	1.102	0.981
Methionine CD^2)^	0.55	0.48	0.45
Cystine^2)^	0.355	0.339	0.318
Methionine CD^3)^	0.562	0.483	0.448
Methionine BD^2)^	0.328	0.303	0.285
Methionine BD^3)^	0.324	0.310	0.291
Calcium^2)^	0.90	0.85	0.80
Non-phytate phosphorus^2)^	0.45	0.42	0.40

CD, control diet; BD, basal diet.

1) Provided (mg/kg diet): thiamin 1; pyridoxine, 2; cyanocobalamine, 0.01; niacin, 15; pantothenic acid, 10; α - tocopherol, 10; riboflavin, 10; biotin, 0.08; menadione, 2; retinol acetate, 2.75; cholecalciferol, 0.06; choline, 650; copper, 8; iron, 45; manganese, 80; zinc, 60; selenium, 0.18; hydrated sodium calcium alumino silicates, 800; phytase, 375 units

2) Calculated.

3) Analyzed.

**Table 2:** Performance of commercial broilers fed different methyl donors in diet containing no supplemental methionine

Treat	BWG (g/b/d)				FI (g/b/d)				FI/BWG			
		
	PS^1)^	S^1)^	F^1)^	1–42 d	PS^1)^	S^1)^	F^1)^	1–42 d	PS^1)^	S^1)^	F^1)^	1–42 d
CD	33.43^A^	67.18^A^	81.71	60.78^A^	35.81^A^	99.16^A^	161.2	98.72^A^	1.073	1.476^B^	1.978	1.624^B^
BD	26.27^C^	53.38^C^	73.86	51.16^C^	30.46^B^	86.76^B^	152.4	89.88^B^	1.162	1.637^A^	2.109	1.759^A^
FA	30.48^B^	54.24^C^	80.07	54.94^B^	34.26^A^	90.37^B^	153.7	92.78^AB^	1.125	1.668^A^	1.928	1.690^AB^
Biotin	29.80^B^	55.93^C^	82.50	56.07^B^	33.12^AB^	93.91^AB^	164.5	97.16^A^	1.113	1.680^A^	1.996	1.732^A^
Vit. B_12_	30.85^AB^	56.14^C^	81.43	56.12^B^	33.82^AB^	93.61^AB^	157.5	94.96^AB^	1.098	1.671^A^	1.940	1.747^A^
Betaine	32.04^AB^	60.86^B^	78.93	57.29^B^	35.98^A^	99.73^A^	164.2	99.98^A^	1.122	1.641^A^	2.092	1.692^AB^
N	10	10	10	10	10	10	10	10	10	10	10	10
P	0.001	0.001	0.230	0.001	0.015	0.012	0.248	0.027	0.061	0.001	0.399	0.001
SEM	0.469	0.891	1.099	0.592	0.510	1.235	1.84	1.002	0.0085	0.0149	0.0236	0.0104

BWG, body weight again; FI, feed intake; CD, control diet; BD, basal diet; FA, folic acid; P, probability; SEM, standard error mean.

1) PS, pre-starter 1–14 d; S, starter 15–28 d; F, finisher 29–42 d.

A–C Means having no common super script in a column differ significantly (p<0.05).

**Table 3:** Slaughter variables (g/kg pre slaughter live weight) in commercial broilers fed different methyl donors in diet containing no supplemental methionine

Treatment	RTC	Breast	Abd fat	Liver
CD	754.4	245.9^AB^	13.26	56.68^C^
BD	742.8	213.4^B^	18.94	68.83^A^
FA (4 mg/kg)	749.2	226.4^AB^	16.64	59.32^BC^
Biotin (1.5 mg/kg)	758.0	220.8^AB^	14.38	61.67^ABC^
Vit. B_12_ (0.1 mg/kg)	750.0	234.0^AB^	16.52	56.57^C^
Betaine, 0.2%	753.7	260.4^A^	17.60	59.48^BC^
N	10	10	10	10
P	0.996	0.030	0.107	0.001
SEM	5.516	4.12	0.711	1.010

RTC, ready-to-cook yield; CD, control diet; BD, basal diet; FA, folic acid; P probability; SEM, standard error mean.

A–C Means having no common super script in a column differ significantly (p<0.05).

**Table 4:** Serum oxidative parameters in commercial broiler fed different methyl donors in diets containing no supplemental methionine

Treatment	Lipid peroxidase (nM MDA/mg protein)	Glutathione peroxidase (U/mL)	RBC catalase (U/g Hb)
CD	1.353^C^	76.29^AB^	586.8^A^
BD	1.671^A^	69.62^B^	396.3^B^
FA (4 mg/kg)	1.690^A^	67.98^B^	375.9^B^
Biotin (1.5 mg/kg)	1.590^B^	79.66^A^	529.5^A^
Vit. B_12_ (0.1 mg/kg)	1.369^C^	79.41^A^	431.5^B^
Betaine (0.2%)	1.431^C^	67.46^B^	539.5^A^
N	10	10	10
P	0.001	0.001	0.001
SEM	0.0223	1.556	15.26

MDA, malonyl dialdehyde; RBC, red blood cells; CD, control diet; BD, basal diet; FA, folic acid; P, probability; SEM, standard error mean.

A–C Means having no common super script in a column differ significantly (p<0.05).

**Table 5:** Immune responses and serum protein fractions in commercial broilers fed different methyl donors in diet containing no supplemental methionine

Treatment	Immune responses		Serum	
	
	LPR	ND titre, log 2	Protein (g/dL)	Albumin (g/dL)
CD	0.635^A^	4.50	2.577^B^	0.522^ABC^
BD	0.393^CD^	4.33	1.737^C^	0.279^D^
FA (4 mg/kg)	0.433^C^	4.50	3.800^A^	0.693^A^
Biotin (1.5 mg/kg)	0.350^D^	4.00	3.072^B^	0.465^BCD^
Vit. B_12_ (0.1 mg/kg)	0.453^BC^	4.00	1.238^C^	0.323^CD^
Betaine, 0.2%	0.497^B^	4.50	2.993^B^	0.592^AB^
N	10	10	10	10
P	0.001	0.863	0.001	0.002
SEM	0.0186	0.1288	0.1542	0.0320

LPR, lymphocyte proliferation ratio; ND, Newcastle disease; CD, control diet; BD, basal diet; FA, folic acid; P probability; SEM standard error mean.

A–D Means having no common super script in a column differ significantly (p<0.05).
